# Longitudinal estimation of intramuscular Tibialis Anterior coherence during subacute spinal cord injury: relationship with neurophysiological, functional and clinical outcome measures

**DOI:** 10.1186/s12984-017-0271-9

**Published:** 2017-06-15

**Authors:** Elisabeth Bravo-Esteban, Julian Taylor, Manuel Aleixandre, Cristina Simón-Martínez, Diego Torricelli, Jose Luis Pons, Gerardo Avila-Martín, Iriana Galán-Arriero, Julio Gómez-Soriano

**Affiliations:** 1 Sensorimotor Function Group, Hospital Nacional de Parapléjicos, SESCAM, Toledo, Spain; 2 Neurorehabilitation Group, Instituto Cajal, CSIC, Madrid, Spain; 3 Toledo Physiotherapy Research Group (GIFTO), Nursing and Physiotherapy Faculty, Universidad de Castilla la Mancha, Toledo, Spain; 4 Stoke Mandeville Spinal Research, National Spinal Injuries Centre, Buckinghamshire Healthcare Trust, NHS, Aylesbury, UK; 5 Harris Manchester College, University of Oxford, Oxford, UK

**Keywords:** Spinal cord injuries, Muscle coherence, Motor recovery, Motor evoked potentials, Neuronal plasticity, Spinal cord injury spasticity

## Abstract

**Background:**

Estimation of surface intramuscular coherence has been used to indirectly assess pyramidal tract activity following spinal cord injury (SCI), especially within the 15-30 Hz bandwidth. However, change in higher frequency (>40 Hz) muscle coherence during SCI has not been characterised. Thus, the objective of this study was to identify change of high and low frequency intramuscular Tibialis Anterior (TA) coherence during incomplete subacute SCI.

**Methods:**

Fifteen healthy subjects and 22 subjects with motor incomplete SCI (American Spinal Injury Association Impairment Scale, AIS, C or D grade) were recruited and tested during 4 sessions performed at 2-week intervals up to 8 months after SCI. Intramuscular TA coherence estimation was calculated within the 10–60 Hz bandwidth during controlled maximal isometric and isokinetic foot dorsiflexion. Maximal voluntary dorsiflexion torque, gait function measured with the WISCI II scale, and TA motor evoked potentials (MEP) were recorded.

**Results:**

During subacute SCI, significant improvement in total lower limb manual muscle score, TA muscle strength and gait function were observed. No change in TA MEP amplitude was identified. Significant increase in TA coherence was detected in the 40–60 Hz, but not the 15–30 Hz bandwidth. The spasticity syndrome was associated with lower 15-30 Hz TA coherence during maximal isometric dorsiflexion and higher 10–60 Hz coherence during fast isokinetic movement (*p* < 0.05).

**Conclusions:**

Longitudinal estimation of neurophysiological and clinical measures during subacute SCI suggest that estimation of TA muscle coherence during controlled movement provides indirect information regarding adaptive and maladaptive motor control mechanisms during neurorehabilitation.

## Background

Limited recovery up to 6–9 months following spinal cord injury (SCI) depends on both the severity and neurological level of the injury [1], especially after motor incomplete injury [1–3]. Currently, clinicians assume that optimal neurorehabilitation can be achieved during subacute incomplete SCI [3], including improvement in gait speed and walking distance [2, 3]. Neurophysiological measures of motor system function provide an objective basis to detect early recovery following SCI [4], although further characterisation of these measures are required, especially in relation to recovery of motor function [5]. Thus, there is a need to perform longitudinal studies of surrogate neurophysiological markers that can be measured quickly and which may be predictive of long-term follow up clinical outcomes. New surrogate measures with standard clinical outcome measures may provide indirect information regarding the development of adaptive neuroplasticity leading to the recovery of motor function [6].

Non-invasive neurophysiological measurement of motor evoked potentials (MEP) allows clinicians to obtain an objective evaluation of the integrity of motor pathways, such as the corticospinal tract, and has been used to predict the severity of SCI [7–9]. Longitudinal studies of corticospinal tract recovery during the subacute phase of SCI using this technique has previously identified a direct relationship between MEP amplitude and gait function [10, 11]. However, the reliability and validity of MEP parameters as biomarkers of recovery after SCI is not clear [12].

Electromyographic (EMG) muscle coherence is a mathematical index that estimates the degree of synchronization of activity from two independent muscle sites or signal sources, calculated in the frequency domain [13]. This non-invasive neurophysiological technique provides indirect information regarding the degree of common neural drive to motor neurones, and has been extensively used to assess pyramidal tract activity in healthy individuals [13–19]. Coherence estimation can be performed on the Tibialis Anterior (TA) [15], with corticospinal motor tract activity inferred from the beta band (10-40 Hz) after SCI, measured either at the low 10-20 Hz [20] or higher 24-40 Hz frequencies [21]. Furthermore, TA coherence estimation from two locations of the same muscle (also called intramuscular coherence estimation) after incomplete SCI subjects has been shown to reflect muscle strength, the severity of injury and the grade of spasticity symptoms [22].

More recently, coherence has been detected in the higher gamma (30-46 Hz) frequency band following SCI recorded from hand muscles in a non-human primate animal model, but without a change in the 15-30 Hz [23]. This fact suggests that higher frequency gamma band coherence could reflect activity mediated from either subcortical or propriospinal systems. However, beta and gamma band EMG coherence have also been related to static and dynamic motor tasks respectively [24]. Further characterisation of muscle coherence above 40 Hz has not been systematically characterised during subacute SCI, especially in relation to beta and standard gamma band activity.

Estimation of TA muscular coherence could help to indirectly identify adaptive neuroplasticity mechanisms related to top-down or spinal motor control, that have been demonstrated in animal models of SCI [25], and which may be present in humans [26]. The hypothesis of this study was that longitudinal estimation of a wider range of gamma-band coherence during the first months of incomplete SCI would provide information regarding the most appropriate test conditions required to highlight motor system neuroplasticity in relation to lower limb motor function and clinical outcome measures. Therefore, the objective of the present study was to monitor muscular TA coherence during controlled foot dorsiflexion in subjects during subacute incomplete SCI, in order to identify significant changes in the overall frequency content in the alpha (5-10 Hz), beta (10-40 Hz) and gamma (40-60 Hz) range, and to relate these changes to improvement in neurophysiological and clinical lower limb motor function. Preliminary findings of this study have been presented as an oral presentation at the International Congress on Neurorehabilitation [27].

## Methods

### Participants and ethical committee approval

This study was conducted at the “Hospital Nacional de Parapléjicos” in Toledo. Healthy subjects were recruited from the Castilla La Mancha region around Toledo. Ethical approval for the study was obtained from the Toledo Clinical Research Ethical Committee (#47, 07/05/2013) and all subjects were required to sign the informed consent before inclusion. The inclusion criteria for the SCI group included: age between 18 to 65 years, more than 1 month from the time of the SCI, injury severity graded between C-D according to the AIS score [28], neurological level between C2 y Th12 with a medically stable evolution and, a TA manual muscle strength score of >2 [29]. The exclusion criteria included: injury to the musculoskeletal or peripheral nervous system, neurological injury to supraspinal centres, epilepsy and pregnancy.

### Experimental design

The experimental design was a descriptive prospective longitudinal design that included 4 repeated testing sessions performed at 2 week intervals during subacute SCI (1st-8th month after injury), which included tests for TA motor evoked potentials (MEP), muscle strength, gait function and lower limb spasticity syndrome. A group of non-injured subjects were recruited in one testing session to obtain reference data (main inclusion criteria, no neurological or musculoskeletal disorders and age between 18 and 65 years old.)

### Experimental procedures

In addition to estimation of TA muscular coherence, maximal voluntary torque (MVT) of ankle dorsiflexion at 10° of plantarflexion, combined with Quadriceps (Q), Hamstrings (H), Triceps Surae (TS) and TA muscle scores performed to assess strength [29], and gait function using the walking index spinal cord injury scale (WISCI II) were measured [30]. Outcome measures related to the SCI spasticity syndrome [31] included muscle hypertonia with the modified Ashworth scale (MAS) [32], and the Penn scale [33]. SCI severity and neurological level was performed by a clinical consultant who had participated in the AIS training course with the European Multicenter Study about Spinal Cord Injury (EMSCI) network. Estimation of TA intramuscular coherence was performed by an engineer who was blinded to the experimental group allocations (MA) [22]. A trained physiotherapist (EBE) assessed neurophysiological outcomes (eg. MEP and TA EMG during controlled foot movement), and clinical measures (eg. muscle strength scores and lower limb spasticity tests).

### Controlled foot dorsiflexion and Tibialis Anterior coherence estimation

The methods used to calculate TA muscular coherence have been published previously [22]. Subjects were seated in an isokinetic dynamometer (KinCom, Chattanooga Group Inc.). The trunk and pelvis were stabilized using straps and the hip, knee and ankle joints were flexed at 90°. A minimal TA muscle score of greater than 2 in the manual muscle score was set as the criteria for recruitment into the study, associated with a moderate muscle contraction level. Testing was performed on the most severely compromised lower limb following SCI, as long as the minimum muscle strength score was met or exceeded. Electromyographic activity was recorded using double differential surface electrodes, with a preamplifier gain of 10 *V*/V and an open filter bandwidth (Delsys Inc. Signal Conditioning Electrodes 3.1). Double differential electrodes were used to reduce electrical cross talk to a minimum between the recording electrodes. To record EMG intramuscular coherence, two surface electrodes were placed on two specific locations on a midline distal and proximal to the mid-point of the TA muscle belly, and separated by a distance of 10 cm to avoid electrical cross-talk [14, 17, 22, 34].

TA muscular coherence was estimated from EMG signals recorded during: i) two maximal isometric dorsiflexion contractions of the TA muscle which were maintained for 5 s, and separated by a 10 s rest period and, ii) ten isokinetic TA muscle contractions with the aim of recording a minimal signal of 3.5 s when combined together (see below), performed at 60°/s and 120°/s, with the ankle joint displaced from 30° plantarflexion to 0° dorsiflexion.

### Tibialis Anterior motor evoked potentials

TA MEP were evoked with a transcranial magnetic stimulation device applied over the primary motor cortex (Magstim Rapid 2, Magstim Company Ltd) [35] with the subject seated on the dynamometer chair as described previously. Participants were provided visual biofeedback to help the subject reach 20% of the maximal dorsiflexion torque. A double-coned coil was oriented onto the cranium to induce a current stimulation in the posterior-to-anterior direction, with the optimal site located by varying the position of the coil from the vertex and increasing stimulus intensity until a MEP in the contralateral TA muscle was recorded at the lowest intensity. Ten MEP were recorded, with the threshold defined as the stimulus intensity at which five MEP of a minimal peak-to-peak amplitude of 100 mV [35].

### Functional and clinical outcome measures

MVT during dorsiflexion was recorded using the isokinetic dynamometer. Individuals were explored in the supine position, including the: i) AIS scale [28]; ii) manual muscle score (0–5) of Q H, TS, TA and total muscle score (0–20 sum up of Q,H,G and TA scores); iii) muscle hypertonia detected during knee and ankle joint passive flexion and extension with the MAS [32]; iv) spasm frequency quantified with Penn scale [33].

### Data analysis

TA electromyographic signals were sampled at a 10 KHz sampling frequency (MicroPlus 1401, Cambridge Electronic Design) and were subsequently down sampled to 2KHz using a low pass filter of 700 Hz to avoid aliasing (Matlab 7.11) [22]. TA intramuscular coherence estimation was performed with the Signal Processing Toolbox of Matlab 7.11 by estimating power spectral densities with Welch’s method [36]. Due to the methodological requirement of estimating coherence from a minimum length of EMG signal, several controlled movement tasks were performed so that when concatenated together a total muscle signal of at least 3.5 s could be analysed for each movement tasks. The signal was divided into 8 data segments using 50% overlapping segments performed with a Hamming window [37].

**Fig. 1:**
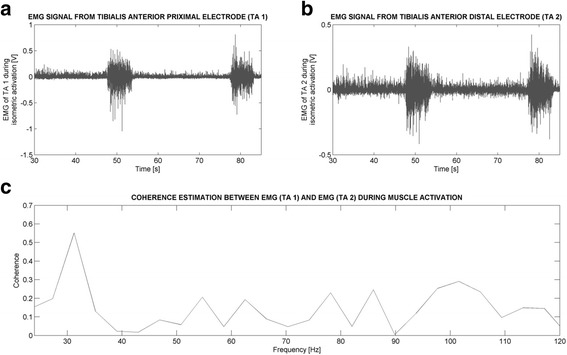
Example Tibialis Anterior intramuscular coherence estimation made during maximal isometric foot dorsiflexion from a subject with SCI and presented for the 0-120 Hz frequency range. The two EMG measurements recorded simultaneously from the two recording sites from the Tibialis Anterior muscle are shown in Panels **a** and **b**. The Tibialis Anterior coherence estimation calculated between them is shown in Panel **c**. A peak in intramuscular coherence can be observed at 30 Hz. Notice that no marked line noise is recorded at 50 Hz. Surface EMG signals were recorded from the midline of the TA muscle, with point 1 located 5 cm proximally (1) and 5 cm distally (2) to the midpoint of the muscle belly

Coherence spectra were calculated against frequency for each subject for quality control and to identify line noise if present (eg. Fig. 1) and for all subjects (see Fig. 4). The coherence estimation for each frequency band (10–16, 15–30, 24–40 and 40-60 Hz) was calculated differently, with all coherence data points within each specific band for each movement averaged to obtain a final grand average coherence value (from 0 to 1). Inspection of individual TA EMG signals were not found to contain a marked level of line noise at 50 Hz, reducing the possibility that this frequency content could have contributed to false positive results associated with intramuscular coherence analysis (see Fig. 1). The velocity dependence of intramuscular TA coherence was calculated by calculating the ratio of its value during isokinetic TA muscle movement at 120°/s and at 60°/s.

Amplified TA MEP were captured using an analogue-digital converter (MicroPlus 1401, Cambridge Electronic Design, UK) for subsequent analysis using a signal processing software package (Signal version 2.14. Cambridge Electronic Design, UK). MEP latency and amplitude were calculated when 7 out of 10 MEP’s were found to be consistent and showed low variability to the mean, with the response averaged from 5 signals. MEP amplitude was calculated from the highest to the lowest multiphase peak. Patients were diagnosed with the spasticity syndrome if a modified Ashworth score > 1 and/or a Penn score of ≥1 was diagnosed in 3 of the 4 of repeated testing sessions [22, 31, 38].

### Statistical analysis

Two commercial software packages (SPSS, version 17.0 and SigmaStat, version 3.1, Systat software, Inc) were used to perform statistical analysis. Kolmogorov Smirnov test confirmed the non-normally distribution of the data and therefore non-parametric statistics were applied. With the exception of spectra data, used for quality data checking, all other data presented here are expressed as median values with 25th and 75th percentiles. In addition, this was performed to facilitate comparison with previous studies made by the group for coherence estimation [22, 27]. The Friedman test was performed for longitudinal analysis within patient groups, followed by application of the Bonferroni post-test used to identify differences among the sessions. The Mann-Whitney test was used to compare TA intramuscular coherence values between the healthy control and SCI groups, and between subjects with and without the SCI spasticity syndrome. Spearman correlations were applied to determine relationships between TA coherence, MEP, neuromuscular parameters (AIS scale, MVT, muscle score and spasticity measures) and gait function (WISCI II). Statistical significance was set at α ≤ 0.05.

## Results

### Subject characteristics

Fifteen healthy subjects (8 males) with a median age of 26 years (24–29, 25th - 75th percentiles value range) and 22 subjects with motor incomplete SCI (18 males) with a median age of 39 years (36–55) were recruited for the follow-up study. No difference was identified for gender.

Initially fourteen subjects with SCI were diagnosed with the spasticity syndrome (modified Ashworth score > 1 and/or a Penn score of ≥1) and 8 were diagnosed without spasticity. However, one of the subjects (from the spastic group) was discharged prematurely after the first session and other individual from the non spastic group failed to complete the fourth session due to secondary complications. Finally, 13 SCI subjects diagnosed with spasticity syndrome and 7 SCI subjects without spasticity completed the study. No difference was identified in general for age, gender, AIS score, and time after SCI for the groups with and without the SCI spasticity syndrome. Furthermore, no significant differences were identified for general clinical characteristics, manual muscle, MVT during dorsiflexion of the foot or WISCI II scores. As expected, the Penn spasm and MAS scores were higher in individuals with SCI spasticity when compared SCI subjects without the spasticity syndrome (*p* < 0.001, Table 1).

**Table 1:** Individual SCI characteristics, and lower limb muscle and spasticity scores measured from the lower limb with the lowest total muscle strength score. Subjects without (1–7) and with the SCI spasticity syndrome (8–20) were recruited into the study

GENDER		AGE	AIS	LEVEL	ETIOL	TIME	TORQUE	Total MS	TA MS	WISCI II	MAS	PENN
1	F	64	C	T12	NT	16	30.0	9	2	8	1	0
2	M	40	D	T11	T	12	63.3	9	3	0	0	0
3	M	36	D	C3	NT	6	83.8	12	3	5	1	0
4	M	30	C	C4	NT	11	89.5	11	3	4	0	0
5	M	58	C	T12	NT	16	109.0	9	4	7	0	0
6	F	36	D	T10	NT	8	132.0	10	4	0	0	0
7	M	52	D	C4	T	14	134.0	15	4	0	0	0
8	M	63	D	C5	T	15	113.0	9	3	4	4	2
9	M	57	C	T6	NT	18	122.3	9	3	3	8	3
10	M	57	C	C6	T	16	26.8	9	2	0	5	1
11	M	25	D	C4	T	15	97.1	10	3	7	2	1
12	M	55	C	C5	NT	8	111.1	11	3	0	0	2
13	M	37	D	C2	T	13	97.4	14	4	8	6	1
14	M	48	D	C4	NT	4	316.0	16	5	20	1	1
15	M	36	D	C5	T	4	124.8	11	2	0	4	3
16	M	33	C	T5	NT	9	200.5	13	4	6	8	2
17	M	46	D	T8	NT	15	96.0	11	3	5	0	1
18	F	37	C	C7	NT	18	34.9	10	3	0	2	1
19	M	34	D	C5	T	7	140.0	13	4	8	5	1
20	M	38	D	T3	T	4	136.0	11	3	0	3	1

M: male; F: female; T: traumatic / NT: non-traumatic; Level: injury level; Etiol: injury etiology; Time: time of first testing session after SCI (weeks). The following variables were recorded during the first test session: Age (years); Torque: dorsiflexion maximum voluntary torque (Nm); Total MS: total muscle strength score (0–20); TA MS (0–5): Tibialis Anterior muscle strength score; WISCI II: Walking Index for Spinal Cord Injury gait score (0–20). MAS: total modified Ashworth score tested during flexion-extension of the knee and ankle joints (0–20); Penn: Penn scale spasm score (0–4)

### Change in clinical and functional scores during subacute SCI

**Fig. 2:**
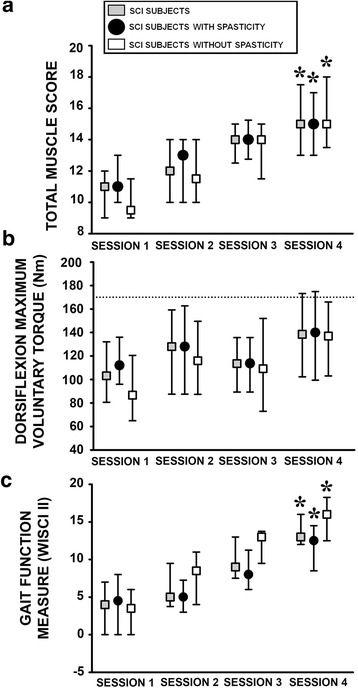
Clinical and functional measures of motor function in all subjects with SCI during the four testing sessions of the subacute phase, and subcategorised into individuals with and without SCI spasticity syndrome. **a**. Total muscle (Quadriceps, Hamstring, Tibialis Anterior and Triceps Surae) strength score (0–20) [29]. **b**. Maximum voluntary dorsiflexion torque. Dotted line represents the median level of torque recorded from the noninjured control group. **c**. Gait function scale (WISCI II) (0–20) [30]. *: *p* < 0.05 with respect to session 1. Data are presented as median values with 25th and 75th percentiles

The median testing period during subacute SCI ranged from 13 weeks (7–15, p25-p75)) for the initial session up to 19 weeks (14–22) for the fourth session, reflecting a repeated testing interval of approximately 2 weeks. Manual muscle strength scores generally increased after first session, for subjects with and without SCI spasticity from session 1 to 4 (*p* < 0.001; Fig. 2a). In contrast, dorsiflexion MVT only showed a specific increase for all subjects with SCI from session 3 (114 Nm 89–136) to 4 (139 Nm, 102–173; *p* < 0.05; Fig. 2b). Gait function also increased during subacute SCI for subjects with or without the spasticity syndrome (Fig. 2c).

### Tibialis Anterior motor evoked potentials

**Fig. 3:**
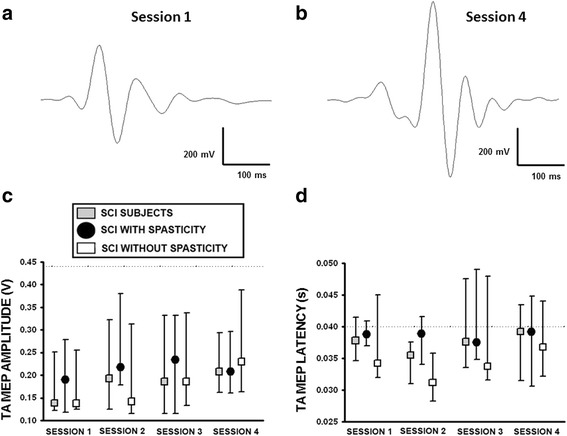
Tibialis Anterior motor evoked potentials recorded during subacute SCI. **a**. Example averaged TA MEP from ten individual records from a subject with SCI performed during session 1, and (**b**.) session 4. **c**. Group averaged TA MEP amplitude and (**d**.) latency recorded in subjects with SCI (*n* = 20), and those diagnosed with (*n* = 13) and without the spasticity syndrome (*n* = 7). Dotted line corresponds to the median of the voluntary control group data. Data are presented as median values with 25th and 75th percentiles

**Fig. 4:**
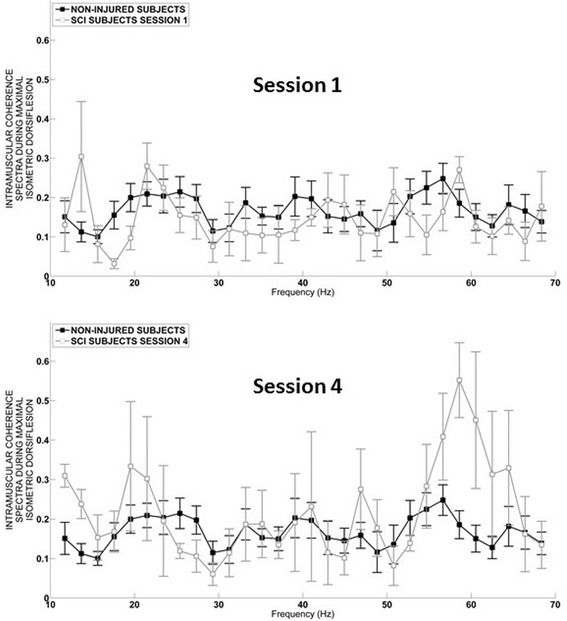
Intramuscular TA muscle coherence spectra (10–70 Hz). Session 1. Mean coherence spectra (as defined in the methods section) was calculated during maximal isometric dorsiflexion in the SCI group (*n* = 20) compared to the non-injured group (*n* = 15) during the first, and Session 2 last testing session 4 (see data analysis section in the methods). Data is presented as mean and standard error

TA MEP records were evoked in subjects with SCI during session 1 (Fig. 3a) and 4 (Fig. 3b). However, no change in TA MEP amplitude (Fig. 3c) or latency (Fig. 3d) was observed during subacute SCI, for individuals with or without spasticity syndrome.

Correlation analysis of TA MEP with clinical and functional motor scores failed to reveal significant relationships with muscle strength scores (Q, H, TA, TS or total score), dorsiflexion MVT or the WISCI II scale, for subjects with or without the spasticity syndrome. However, the SCI spasticity group were characterised by correlations between TA MEP amplitude and the Penn spasm scale (rho = 0.41; *p* = 0.03), and TA MEP latency with TS muscle (rho = 0.33; *p* = 0.01) and total MAS score (rho = 0.28; *p* = 0.04).

### TA muscle coherence during controlled activation

**Fig. 5:**
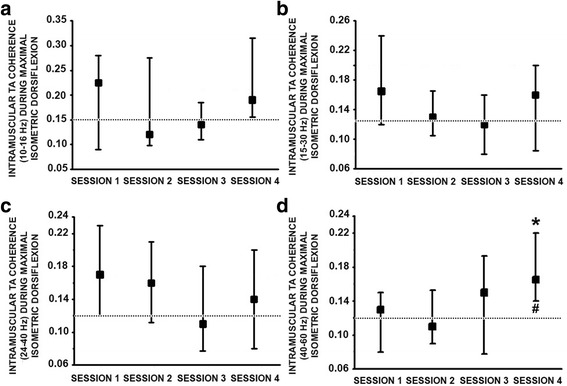
Intramuscular TA coherence estimated following incomplete SCI during the 4 repeated testing sessions. **a**. Median TA coherence analysed within the 10–16 Hz, **b**. 15–30 Hz, **c**. 24–40 Hz and **d**. 40–60 Hz frequency range during maximal isometric dorsiflexion from 20 subjects with SCI. Dotted line corresponds to the median non-injured group coherence value. *: *p* ≤ 0.05 with respect to session 1 and #: *p* ≤ 0.05 with respect to the non-injured control data. Further methodological information can be found in the analysis section of the methods. Data presented as median values with 25th and 75th percentiles

Analysis of the entire 10-60 Hz TA coherence bandwidth, recorded during maximum isometric dorsiflexion in the non-injured and SCI groups, showed no marked difference during the first testing session for individual frequency (Fig. 4 – Session 1). In contrast, during the last session a significant increase in the 50-60 Hz bandwidth was evident for subjects with SCI (Fig. 4 – Session 4). At the group level this increase in TA muscle coherence during subacute SCI was evident as a significant increase in the 40-60 Hz bandwidth during MVT dorsiflexion (*p* = 0.03, Fig. 5d), but not within the other studied bandwidths either during isometric (Fig. 5a-c) or for isokinetic movement (10-60 Hz). Furthermore, a weak correlation was observed between the 40-60 Hz TA coherence bandwidth measured during MVT dorsiflexion and the TA muscle score (Rho = 0.22, *p* = 0.05).

With respect to SCI severity, subjects diagnosed with an AIS D injury were characterised with higher TA coherence within the 24-40 Hz, but not 10–16 Hz, 15–30 Hz, and 40-60 Hz bandwidths, when compared to the AIS C group, tested during isometric MVT for the entire subacute period (0.16, 0.11–0.19 vs 0.11, 0.09–0.15 respectively, *p* = 0.03).

### TA muscle coherence and SCI spasticity syndrome

**Fig. 6:**
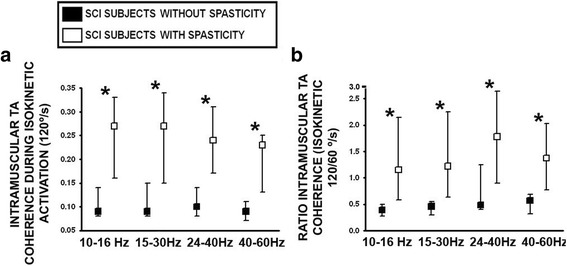
Velocity-dependent intramuscular TA coherence estimated during the last testing session of subacute SCI, in subjects diagnosed with and without the spasticity syndrome. **a**. Intramuscular TA coherence was estimated during isokinetic dorsiflexion of the foot at 120°/s, and (**b**.) expressed as a ratio of 120/60 °/s movement in subjects with and without SCI spasticity syndrome. Ankle joint movement was set at the same angular displacement for all subjects (see methods). *: *p* ≤ 0.05

During the last testing session of subacute SCI (Session 4) intramuscular TA coherence estimation at high isokinetic movement speeds (120°/s) was higher for subjects with the SCI spasticity syndrome for all the studied bandwidths (Fig. 6a, 40-60 Hz: *p* < 0.01; 24-40 Hz: *p* < 0.01; 15-30 Hz: *p* = 0.02 and 10-16 Hz: *p* = 0.04). Likewise, the ratio of TA intramuscular coherence measured during isokinetic movement at 120/60 °/s also revealed higher values for the SCI spasticity group (Fig. 6b). Correlation analysis also revealed a negative relationship between velocity-dependent TA 40-60 Hz muscle coherence and specific SCI spasticity symptoms, specifically with the total lower limb (rho = −0.63; *p* = 0.02) and knee flexion MAS score (rho = −0.70; *p* = 0.01).

## Discussion

For the first time, this study shows that limited recovery of lower limb muscle strength and general walking function during the first few months after incomplete SCI is accompanied by a significant increase in high-frequency (>40 Hz) band intramuscular TA coherence, without an increase in either TA motor evoked potential amplitude or beta band 15-30 Hz intramuscular coherence. Increase in high-frequency (>40 Hz) TA muscle coherence without an increase in TA MEP amplitude suggests that subcortical motor control mechanisms could play a role in the early recovery of lower limb muscle function during subacute SCI. Furthermore, the development of SCI spasticity was associated with a lower level of 15-30 Hz intramuscular TA coherence and TA motor evoked potentials, which highlights the relationship between this motor syndrome and corticospinal tract dysfunction.

### Beta and gamma intramuscular TA coherence after spinal cord injury

Limited recovery of lower limb function, such as gait, observed in animal models of SCI has been shown to reflect neuroplasticity and new connections between corticospinal, reticulospinal and propriospinal motor control mechanisms [25]. Indeed, recovery of motor function in humans following incomplete SCI most probably includes similar neuroplasticity mechanisms active throughout the motor neuroaxis [1, 39, 40]. In the current study subjects with SCI showed clinical motor improvement, in addition to an increase in intramuscular gamma band TA 40–60 Hz coherence, but not in the beta band, which has traditionally been associated with corticospinal activity as assessed with transcraneal magnetic stimulation [41]. Intramuscular coherence within the gamma band has been proposed to reflect reticulospinal and propriospinal residual activity after spinal injury and other pathologies [23, 41, 42], although the contribution of corticospinal activity to standard gamma band coherence cannot be excluded [43, 44]. Interestingly in a previous study we have shown that TA gamma band coherence in motor incomplete SCI patients correlates with severe spasticity symptoms, including spasms and hypertonia during isometric dorsiflexion [22]. Taken together, these studies suggest that increased activity within reticulospinal and/or propriospinal control mechanisms, without an increase in TA MEP (see below), could be mediated both adaptive and maladaptive neuroplasticity of subcortical motor control pathways after SCI [45].

In our previous study, estimation of TA beta band coherence (15-30 Hz) after SCI was shown to correlate with residual muscle strength and gait function [22]. However, in the present study, 15–30 Hz coherence was generally stable in individuals with incomplete SCI and showed no change during subacute SCI. One explanation could be the relatively high number of subjects with the SCI spasticity syndrome (14/22), which may have blunted adaptive neuroplasticity of the corticospinal motor control system [22]. Another explanation could be that estimation of coherence activity in the 24–40 Hz may better reflect corticospinal activity after incomplete SCI [21], especially as we have shown previously that this band correlates with dorsiflexion isometric torque and is higher in patients with less severe SCI (AIS D) [22]. Nevertheless, no change in 24-40 Hz was detected in the present study for our cohort during subacute SCI.

Motor evoked potentials elicited by transcranial magnetic stimulation permits an objective assessment of SCI severity [8, 9, 46, 47], although the reliability of this technique in smaller patient cohorts has been questioned, especially in relation to reliability [12]. In other larger studies MEP amplitude and latency have been shown to correlate with functional and clinical motor outcome measures in specific cohorts [7, 46]. In the present study, TA MEP amplitude was decreased respect to the non-injured group, measured during subacute SCI, suggesting that longer follow-up times may be required to identify correlations with intramuscular TA coherence [11].

### SCI spasticity syndrome and intramuscular coherence after spinal cord injury

The complex pathophysiology of the SCI spasticity syndrome may involve concomitant changes in cortical, subcortical and spinal motor control pathways [48, 49]. In this study, spasticity in subjects with SCI was characterised by a general decrease in intramuscular beta band TA coherence (15–30 Hz) during the isometric dorsiflexion. This reduction in potentially cortical motor control activity, together with correlations between TA MEP and severity of spasticity symptoms may suggest that maladaptive neuroplasticity of the corticospinal pathway plays a role in the development of this motor disorder.

In general, no difference was found with either the clinical scores such as the AIS, manual muscle, maximal voluntary dorsiflexion torque and gait scores, or the neurophysiological (TA muscle coherence or MEP’s) measures when they were compared between patients with and without spasticity during subacute SCI. However when TA coherence was measured for all the frequency bands studied (10–60 Hz), a significant velocity-dependent increase was noted for the SCI spasticity group, suggesting a generalised excitability of activity within the neuroaxis [22] including subcortical motor control systems [45]. Furthermore gamma intramuscular TA coherence was also higher at faster isokinetic speeds, but also correlated negatively with muscle hypertonia, similar to our previous study [22]. Finally, because low frequency coherence has been associated with activity in spinal networks (10-16 Hz) [21], increased TA coherence detected during isometric activation in subjects with spasticity suggests that hyperexcitability of spinal motor control mechanisms also play a role during faster movements.

### Limitations

Due to the small cohort size (*n* = 20) and limited follow up to 6 weeks during subacute SCI, caution should be made when we conclude that the increase in TA muscular coherence within the high-frequency (>40 Hz) band is not due to a corresponding increase in TA MEP amplitude. Longer follow up would also have clarified the impact of the development of SCI spasticity on TA muscle coherence. Furthermore, the relatively small and short duration TA muscle activation produced by patients with SCI may have affected the size of MEP, suggesting that standardisation of the TA MEP should be made to avoid methodological bias.

## Conclusions

This study demonstrates that periodic estimation of high frequency intramuscular gamma band TA coherence (>40 Hz) in subjects with subacute incomplete SCI, may provide additional diagnostic information regarding the development of either adaptive or maladaptive motor control neuroplasticity during lower limb motor recovery. More information is required regarding the involvement of subcortical mechanisms that could mediate the limited lower limb motor recovery at this stage of rehabilitation, particularly as no evidence for a significant increase in either TA motor evoked potentials or 15-30 Hz beta band coherence identified in this small patient cohort. An important set of experimental studies using animal models have identified several secondary mechanisms of neuroplasticity organised at the subcortical and intraspinal level that could mediate adaptive neuronal motor control mechanisms following incomplete SCI, which suggest that the present clinical findings could be mediated by similar mechanisms such as the reticulospinal and/or propiospinal tracts. At the clinical level, evidence-based diagnosis of motor control adaptive neuroplasticity could be achieved by periodically testing TA intramuscular coherence during a simple motor task, but further studies should be performed to assess the clinical impact of this technique at the individual level.

## Abbreviations

AIS: American Spinal Injury Association Impairment Scale
EMG: electromyographic activity
H: Hamstrings
MAS: modified Ashworth scale
MEP: motor evoked potentials
MVT: Maximal voluntary torque
Q: Quadriceps
SCI: spinal cord injury
TA: Tibialis Anterior
TS: Triceps Surae
